# A new analytical solar radiation pressure model for current BeiDou satellites: IGGBSPM

**DOI:** 10.1038/srep32967

**Published:** 2016-09-06

**Authors:** Bingfeng Tan, Yunbin Yuan, Baocheng Zhang, Hou Ze Hsu, Jikun Ou

**Affiliations:** 1 State Key Laboratory of Geodesy and Earth’s Dynamics, Institute of Geodesy and Geophysics, Wuhan 430077, China

## Abstract

An analytical solar radiation pressure (SRP) model, IGGBSPM (an abbreviation for Institute of Geodesy and Geophysics BeiDou Solar Pressure Model), has been developed for three BeiDou satellite types, namely, geostationary orbit (GEO), inclined geosynchronous orbit (IGSO) and medium earth orbit (MEO), based on a ray-tracing method. The performance of IGGBSPM was assessed based on numerical integration, SLR residuals and analyses of empirical SRP parameters (except overlap computations). The numerical results show that the integrated orbit resulting from IGGBSPM differs from the precise ephemerides by approximately 5 m and 2 m for GEO and non-GEO satellites, respectively. Moreover, when IGGBSPM is used as an a priori model to enhance the ECOM (5-parameter) model with stochastic pulses, named ECOM + APR, for precise orbit determination, the SLR RMS residual improves by approximately 20–25 percent over the ECOM-only solution during the yaw-steering period and by approximately 40 percent during the yaw-fixed period. For the BeiDou GEO01 satellite, improvements of 18 and 32 percent can be achieved during the out-of-eclipse season and during the eclipse season, respectively. An investigation of the estimated ECOM D0 parameters indicated that the β-angle dependence that is evident in the ECOM-only solution is no longer present in the ECOM + APR solution.

Following the completion of the first deployment phase, which involved a constellation consisting of 5 satellites in geostationary orbit (GEO), 5 in inclined geosynchronous orbit (IGSO), and 4 in medium earth orbit (MEO), the Chinese BeiDou Navigation Satellite System (BDS) has been officially providing continuous passive positioning, navigation and timing services for users throughout the Asia-Pacific area since December 27, 2012. In 2014, during the 94^th^ meeting of the International Maritime Organization (IMO) Maritime Safety Committee, IMO’s recognition of BDS made it the third navigation satellite system serving global maritime users, after the GPS and GLONASS systems. On March 30^th^, 2015, China’s first New-Generation BeiDou Navigation satellite (an IGSO), known as the 17^th^ BeiDou satellite, was successfully launched, signalling the beginning of BDS’ expansion from regional to worldwide coverage. Five additional similar satellites, each with a new navigation signal system, inter-satellite links, and other test features, were launched during the second half of 2015, bringing the satellite navigation system closer to completion.

With the increasing quality requirements for civilian and scientific applications based on the emerging BDS^1^,^2^,^3^,^4^, achieving precise orbit determination for the constellation has become one of the most challenging issues facing the Global Navigation Satellite System (GNSS) community^5^. At present, the orbit determination performance for BeiDou remains inferior compared with that of other GNSS constellations, such as GPS and GLONASS. This problem has initially been attributed to the lack of a well-distributed worldwide tracing network, the lack of official satellite and receiver antenna offset and variation parameters, the difficulty of integer ambiguity resolution, the poor geometric distribution of the satellites, or the frequent manoeuvring of the GEO satellites. Recently, evidence of deficiencies in the solar radiation pressure (SRP) modelling was found in investigations of the BeiDou satellite SRP modelling and attitude control modes. The force from SRP is the largest perturbation acting on GNSS satellites after the gravitational attraction from the Earth, Sun, and Moon, making it the largest source of error in the modelling of GNSS orbital dynamics. To address this problem, the current study focuses on solar radiation modelling for all types of BeiDou satellites.

Many previous studies have considered the problem of precise orbit determination for BeiDou satellites. Generally, four classes of SRP models have been applied to BeiDou satellites. In SRP models of the first type, the traditional Empirical CODE Orbit Model (ECOM) of the Center for Orbit Determination in Europe (CODE) is used, with no a priori model and no additional constraints^6^,^7^,^8^,^9^. In particular, Liu, J. *et al*.^7^ applied a four-step analysis to determine the optimized and smallest subset of the ECOM 9 SRP model for BeiDou GEO satellites and proposed a new empirical SRP model called BGSM, which includes three constants and three periodic terms (sine terms in the D and X directions and a cosine term in the Y direction), for BeiDou GEO satellites. These studies have shown that the root mean square (RMS) of the orbit overlap during the out-of-eclipse season can reach 1–2 decimetres for MEO/IGSO satellites and several decimetres for GEO satellites. In SRP models of the second type, a relatively simple analytical SRP model is utilized to enhance ECOM^10^,^11^. These studies have demonstrated SLR validation of better than 30 centimetres for IGSO satellites during yaw manoeuvres. In the third class of SRP models, additional constraints are applied to enhance ECOM^12^,^13^,^14^. These studies have demonstrated RMS orbit overlap comparison accuracies of approximately 20 centimetres for MEO/IGSO satellites and several metres for GEO satellites. The SLR residuals are better than 10 centimetres for MEO/IGSO satellites and several decimetres for GEO satellites. In the last class of SRP models, analytical models for the BeiDou satellites are developed. Feng *et al*.^15^ derived a similar ROCK SRP model for BeiDou satellites based on a ray-tracing method, but this model is available only for IGSO satellites, and no analysis of precise orbit determination with the model for BeiDou satellites has been performed.

In the absence of a detailed, structure-based analytical SRP model for current BeiDou satellites, the ECOM SRP model without a priori information was used for precise orbit determination for BeiDou in these previous works. ECOM was initially developed by CODE in the early 1990s for GPS satellites in yaw-steering mode, based on many years of observations of and orbit products from GPS satellites. Unlike GPS satellites, the BeiDou satellites have different body shapes. The BeiDou IGSO/MEO satellites operate in the yaw-fixed mode when the Sun’s elevation angle with respect to the satellite orbital plane, β, is less than 4° and the yaw angle is less than 5°, which occurs once every half a year and lasts for 8–15 days, whereas the BeiDou GEO satellites always operate in the yaw-fixed mode. Obviously, ECOM currently has difficulty modelling the orbits of the BeiDou satellites to sufficient precision. Therefore, it would be advantageous to develop a more appropriate SRP model for all types of BeiDou satellites.

SRP can be modelled using empirical, semi-empirical or analytical methods^16^,^17^,^18^,^19^,^20^,^21^,^22^,^23^,^24^,^25^,^26^. Currently, high-accuracy orbit determination at the analysis centres of the International GNSS Service (IGS) relies on empirical SRP models, such as ECOM and the GPS Solar Pressure Models (GSPM), and semi-empirical SRP models, such as the adjustable box-wing model. However, both types of models rely upon long-term observations from an enormous number of tracking stations and long-term precise orbit products from the GNSS constellation, both of which are unfortunately unavailable early in the operation of a GNSS. Regarding the BDS constellation, a relatively small number of ground tracking stations have been deployed and a limited amount of precise orbit products is currently available, thus motivating the development of an analytical solar radiation model.

Our purpose is to achieve a better understanding of how the physical properties of a satellite, such as its shape, mass, and attitude control mode and the optical properties of its surfaces, affect its trajectory and to develop an analytical SRP model for the current BeiDou satellites that can be used as an a priori model to augment the empirical ECOM for precise orbit determination for BeiDou satellites. For readability, we provide a glossary of the terms and abbreviations used in this paper in Supplementary Note S1.

## Methods

### A set of proposed analytical SRP models (IGGBSPM) for BeiDou GEO, IGSO and MEO satellites

We use a ray-tracing method^27^ similar to that used in the development of the G1A and G2A models^28^ for the GLONASS satellites. We describe the BeiDou satellites as having a box-wing-shaped structure combined with several other major components, i.e., the spacecraft surface is decomposed into a box-shaped body, solar panel wings and several other major components. A pixel array is used to simulate the light from the Sun. Each pixel is converted into a ray, and the trajectory of each ray is then calculated to determine which parts of the spacecraft are sunlit and which are in shadow. After this process has been completed for all pixels, the accelerations due to each ray are added together. Analytical SRP models are presented here for BeiDou GEO, IGSO and MEO satellites based on the proposed method and the physical properties of the three types of satellites, including the satellites’ shapes, masses, and attitude control modes and the optical properties of their surfaces. The method is described in detail in the following.

### Coordinate system and yaw attitude profile for GNSS/BeiDou satellites

The attitude of a GNSS satellite can be described by introducing a spacecraft-body-fixed right-handed Cartesian system. The system is defined as follows: the origin of the system is located at the satellite centre of mass; the Z axis is parallel to the GNSS navigation signal antennae, which is also called the satellite antenna boresight; and the Y axis is parallel to the solar panels of the satellite. The X axis completes the right-handed coordinate system; the +X direction is always oriented towards the Sun for GPS BLOCKII/IIA, IIF, BeiDou and GLONASS-M satellites and away from the Sun for GPS BLOCKIIR satellites.

The nominal attitude of a GPS satellite should satisfy two requirements. First, the Z axis, which is parallel to the GPS navigation signal antennae, should always point towards the geocentre of the Earth; second, the Y axis, which is the rotational axis of the solar panels, should always be perpendicular to the satellite-Sun direction. The nominal attitude is maintained with the assistance of Earth sensors and Sun sensors and is adjusted by the momentum wheel mounted on board. As a result, the GPS satellite continuously rotates about the Y and Z axes, operating in a mode called the yaw-steering mode. However, as the angle of the Sun above the orbital plane grows small, the satellite’s yaw-manoeuvring rate considerably increases; thus, the nominal attitude of the GPS satellite may not be properly maintained. Hence, the GPS satellite experiences a so-called eclipse season. Because of the limitations imposed by the maximum hardware yaw rate, the GPS satellite will undergo noon-turn and midnight-turn manoeuvres. When the Sun sensors lose sight of the Sun, the GPS satellite will undergo shadow-crossing manoeuvres.

**Figure 1:**
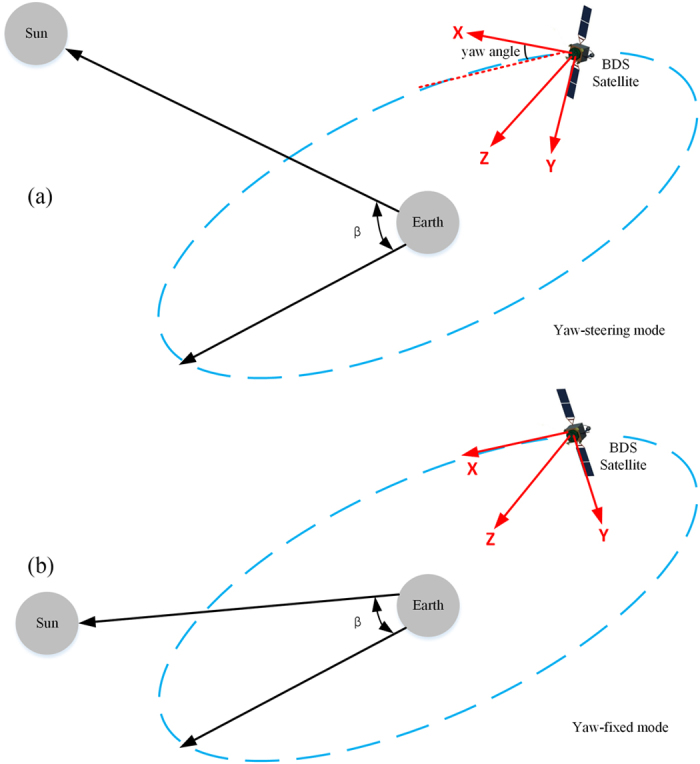
BDS body-fixed coordinate system and attitude modes. (**a**) Yaw-steering attitude mode. (**b**) Yaw-fixed attitude mode.

Unlike GPS satellites, BeiDou IGSO/MEO satellites use a yaw attitude profile similar to that of the Japanese Quasi Zenith Satellite System (QZSS)^29^, which consists of a yaw-steering mode and a yaw-fixed mode, depending on the Sun’s elevation angle above the orbital plane. The literature to date reports that the yaw attitude switches between the yaw-steering mode and the yaw-fixed mode when the Sun’s elevation angle with respect to the satellite orbital plane, |β|, is close to 4°^10^. In the yaw-fixed mode, the yaw angle is always fixed at zero and the solar panel axis is oriented normal to the orbital plane. As a result, the solar panels are not strictly perpendicular to the satellite-Sun direction but rather have a slight misalignment angle, which introduces a so-called Y-bias force. When a BeiDou satellite is in the yaw-steering mode, the +X surface is always exposed to radiation from the Sun, whereas the +Z and −Z surface are alternately exposed. By contrast, when the satellite is operating in the yaw-fixed mode, the ±Y and –X surfaces are also exposed to the Sun; In the near future, this phenomenon should be incorporated into thermal re-radiation modelling. Moreover, it should be noted that BeiDou GEO satellites are always kept in the yaw-fixed mode. The BDS body-fixed coordinate system and the attitude modes are illustrated in Fig. 1.

### Analytical SRP modelling for BeiDou GEO, IGSO and MEO satellites

To describe the SRP force, we consider a single flat surface of area A that is subjected to an incident photon flux at an inclination angle 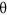. According to Einstein and Planck’s theories regarding relativity and electromagnetic radiation, when a photon is absorbed by a surface, the momentum transferred to the material per unit area per unit time will be E/c, where E is treated as a constant in this study (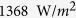). Let the reflectivity and specularity coefficients of the material be 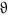 and 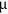, respectively. Then, the force induced by the SRP can be resolved into normal (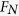) and shear (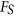) components and can be considered to consist of three contributions: the force due to direct radiation, the force due to specularly reflected light and the force due to diffusely reflected light.

The force due to direct radiation is given by


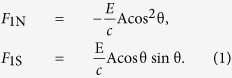


The force due to specularly reflected light is given by


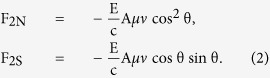


Under the assumption that the absorbed radiation is consistent with Lambert’s law, the force in the normal direction due to diffusely reflected light can be expressed as


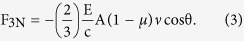


The total radiation pressure force on the single flat surface of area A is finally obtained as follows:


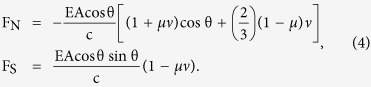


The above equations^27^ establish the basis for the analytical solar radiation modelling for BeiDou satellites. In this study, we use a ray-tracing method similar to a previously published method^27^ that was used to develop the G1A and G2A models for the old GLONASS IIv satellites and GPS BLOCKIIR satellites.

The second-generation BDS satellites that are currently in orbit were designed based on the DongFangHong-3A (DFH-3A) satellite platform (http://www.cast.cn/Item/Show.asp?m=1&d=2875), which possesses a cuboid structure. Information regarding the mass, the dimensions of the satellite bus and solar panels, and the optical properties of each component of the spacecraft (such as absorptivity, reflectivity and specularity) were obtained from the satellite manufacturer (China Academy of Space Technology) through official cooperation and cannot currently be disclosed.

First, we describe the modelled BeiDou satellite as a box-wing-shaped structure combined with several other major components. Each of the components is decomposed into several regular or irregular planar polygons and several cylindrical components.

Second, the photon flux from the Sun is gridded into an array of rectangular pixels with a resolution of 10 mm in a plane orthogonal to the satellite-Sun direction. Each pixel is converted into a ray, and the trajectory of each ray is then calculated to determine which parts of the spacecraft are sunlit and which are in shadow. The illuminated parts of the components are treated as starting points for new rays scaled by the specularity of the material. After this process has been completed for all pixels, the accelerations due to each ray are added together.

Third, the angle between the Earth and Sun directions as seen from the satellite (called the EPS angle) is chosen as the angular variable in the model. The initial pixel array is generated at an EPS angle of 0 degrees, and and the pixel array is rotated with a step size of 10 degrees to calculate the SRP acceleration on the satellite at EPS = 0, 10, 20, 30, 40, …, 360 degrees. Thus, a set of SRP accelerations on the X and Z axes of the satellite is generated at each unique EPS angle.

**Figure 2:**
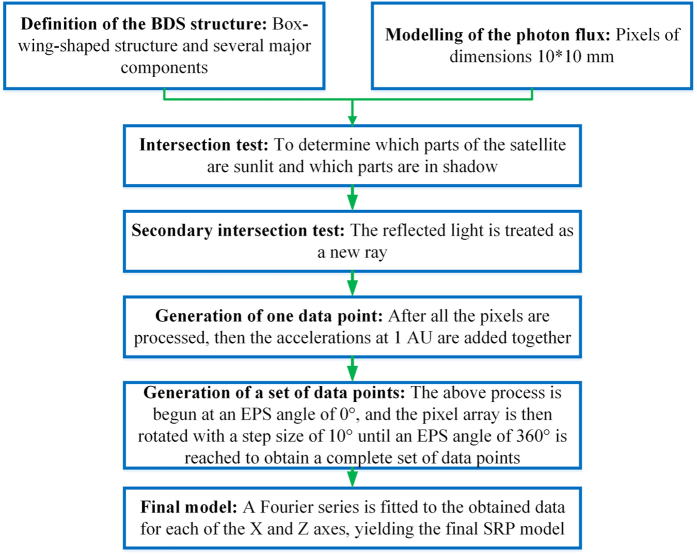
Flowchart of the SRP modelling procedure for BDS.

Finally, a Fourier series is fitted to the data obtained using the process described above for the X and Z axes. For each of the two axes, m data points are generated as follows:


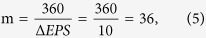


where Δ*EPS* is the EPS step size and m is the number of obtained data points. This enables the calculation of a Fourier series to order n = 17. The final series for the X and Z axes are written in the conventional Fourier format as follows:


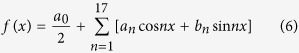


where x is the EPS angle and *a*_*n*_ and b_*n*_ are the Fourier coefficients. Hence, when the positions of the BeiDou satellite, the Earth, and the Sun at a unique epoch are known, the above series can be used to calculate the SRP acceleration. Because the model is calculated at 1 AU (astronomical unit), the user should scale f(x) by the square of 1 AU divided by the square of the satellite-Sun distance at the required epoch. The proposed analytical solar radiation model is named IGGBSPM, which is an abbreviation for Institute of Geodesy and Geophysics BeiDou Solar Pressure Model. The Fourier coefficients of IGGBSPM for the BeiDou GEO, IGSO and MEO satellites are given in Tables S1, S2 and S3. The SRP modelling procedure for BDS is summarized in Fig. 2.

## Results

Many metrics may be considered for assessing the performance of the SRP model developed in this study. Unfortunately, overlap computations are not useful indicators of the SRP model’s accuracy. The errors introduced into a CODE-only solution by the neglect of the IGGBSPM contribution are strongly correlated between one day and the next and exert little effect on the overlap statistics^28^,^30^. We therefore refrain from presenting overlap computations in the context of the present work, instead preferring to focus on more suitable performance metrics, such as numerical integration, SLR residuals and analyses of empirical SRP parameters. BeiDou satellites G01, I03, I05 and M03, which are equipped with laser retro-reflector arrays, were chosen as the basis of a numerical integration test and a precise orbit determination test. The numerical integration results and the SLR residual results after precise orbit determination using the ECOM SRP model with and without enhancement with the analytical SRP model developed in this study are evaluated separately in the following sections.

### Numerical integration

In theory, if the dynamic models used below, especially the SRP model, are sufficiently accurate, then an accurate orbit can be generated via integration. Daily numerical integration of the trajectory for each of the BeiDou satellites G01, I03, I05 and M03 was performed for the entire year of 2014. For each satellite, the integration test covered a complete interval during which the Sun’s elevation angle with respect to the satellite orbital plane, β, varied between its minimum and maximal values. The dynamic models used for integration are summarized in Table S4.

Weekly Multi-GNSS precise satellite orbit products generated by the International GNSS Monitoring & Assessment System (iGMAS) Analysis Center at our institute were taken as the initial conditions for orbit integration. The initial Earth-centred, Earth-fixed (ECEF) state vector was transformed into the J2000 Earth-centred inertial (ECI) frame using a nine-parameter rotation matrix. After the integration process, the orbit was transformed back to the ECEF frame and compared with the weekly precise orbit, and the daily orbit RMS residuals of the along-track, cross-track and radial components were calculated for each satellite. Then, the numerical integration accuracy of the analytical SRP model proposed in this study was evaluated based on the calculated residuals.

The residuals were calculated by taking the difference between the integrated and precise trajectories at each epoch represented in the precise orbits; the daily RMS residuals for each arc of BeiDou satellites G01, I03, I05 and M03 are shown in Figure S1 and summarized in Table 1. This table shows that the RMS residuals for the four satellites are less than 1 metre in the radial component and less than 2 metres in the cross-track component. In addition, the along-track component has the largest magnitude, with values of approximately 2 metres for satellites I03, I05 and M03 and 5 metres for G01. Meanwhile, Figure S1 shows that when the Sun’s elevation angle with respect to the satellite orbital plane, β, varies from −4° to 4°, that is, for Days of Year 256–272 for G01, Days of Year 178–188 for I03, Days of Year 204–218 for I05 and Days of Year 184–194 for M03, a significant jump occurs. The peak amplitudes of the along-track component reach 7.3 m, 4.2 m, 4.6 m, and 4.5 m for satellites G01, I03, I05, and M01, respectively, during the yaw-fixed period. These findings clearly indicate the existence of some force in the yaw-fixed case that is not modelled in the SRP model. We previously noted that BeiDou IGSO/MEO satellites switch to yaw-fixed mode at low Sun elevations, and in this mode, the solar panels are not completely orthogonal to the Sun-satellite direction, which can induce a significant Y-bias perturbing the satellites. It is clear that we must consider this Y-bias in the yaw-fixed case.

According to Ziebart, M. *et al*.^27^, if the solar panel misalignment angle (φ) is small, then the perturbing force exerts a second-order effect with respect to the force parallel to the Sun-satellite direction. Moreover, the Y-bias force is linearly related to φ. The Y-bias force is derived as follows^27^:


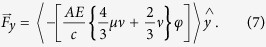


As the misalignment angle in yaw-fixed mode is expected to be limited to no more than 5° according to the BDS operation control strategy, we model the Y-bias as a constant perturbing force when the GEO/IGSO/MEO satellites are operating in yaw-fixed mode. The orbit integration results for satellites I03, I05 and M03 with the inclusion of the additional Y-bias force during the yaw-fixed period and for satellite G01 during the eclipse season are shown in Figure S2. It can be seen that by adding a constant Y-bias force during the yaw-fixed period, a significant improvement can be achieved in the orbital accuracy attained through orbit integration; specifically, for the along-track component, improvements of 26, 26, 21 and 18 are achieved for satellites G01, I03, I05 and M03, respectively. Therefore, during post-processing, the Y-bias force acting on BeiDou satellites during the yaw-fixed period should be taken into account.

### Precise orbit determination for BeiDou satellites

To further assess the SRP model developed in this study, we used it as an a priori model for precise orbit determination for BeiDou satellites. Two solutions were generated: the 5-parameter ECOM-only solution and the solution obtained using the SRP model proposed in this study as an a priori model to enhance the 5-parameter ECOM solution (called ECOM + APR in this study). The SLR residuals for the two solutions were analysed to evaluate the accuracy of the BeiDou satellite orbits generated by the two solutions.

### Stations, observations and the dynamic model

**Figure 3:**
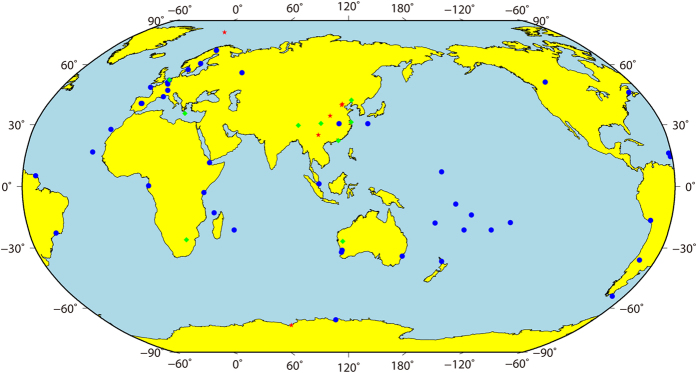
Tracking stations used in precise orbit determination for BeiDou satellites. The tracking stations are represented by red five-pointed stars, green circles and blue circles for iGMAS, BETS and MGEX stations, respectively. This figure was produced using the GMT software package^36^ (version number: 4.5.8, URL: http://gmt.soest.hawaii.edu/).

Observation data from iGMAS, The Multi-GNSS Experiment (MGEX) and the BeiDou Experimental Tracking Stations (BETS) network for Days of Year 117–305 in 2014 were processed. In total, the processed data included observations from 45 MGEX stations, 9 iGMAS stations and 9 BETS stations. A map of the distribution of the selected stations from the iGMAS, MGEX and BETS tracking networks for BeiDou orbit determination is shown in Fig. 3.

We incorporated the SRP model developed in this study into the routine software package that has been developed by the Analysis Centre at the Institute of Geodesy and Geophysics as an a priori SRP model for BeiDou satellites. The ECOM-only solution was obtained, and the SRP model developed in this study was then applied as an a priori model to enhance the ECOM solution; finally, the results from the two solutions were compared through an analysis of their SLR residuals. Important aspects of the chosen processing strategy with regard to the observation model and the force model are listed in Table S5 31,32.

### SLR residual statistics

SLR is a powerful and independent technique that enables independent validation of satellite orbits. The difference between the observed SLR values and the distances computed using the BeiDou satellite orbits and the SLR reference station coordinates can be used to identify whether systematic biases or unexpected errors exist in the estimated BeiDou satellite orbits.

BeiDou satellites G01, I03, I05 and M03 are monitored by the International Laser Ranging Service (ILRS) SLR tracking stations. The SLR range observations can be accessed through ftp services provided by the ILRS. For this study, the latest SLR station coordinates and system eccentricities were obtained from the ILRS. The retro-reflector offsets of the BeiDou satellites were also obtained from the ILRS. There are 531, 937, 1160 and 1310 normal points (NP) available for satellites G01, I03, I05 and M03, respectively, during the study period. These NPs are from the Yarragadee, Changchun, Zimmerwald, Shanghai, Mount Stromlo, Graz, Herstmonceux, Grasse, Matera and Wettzell stations. The SLR observations were corrected using the retro-reflector offsets provided by the ILRS, and several bad NPs were removed after detection and comparison. The yaw-steering and yaw-fixed cases were evaluated separately for the four BeiDou satellites. The results are summarized in Tables 2, 3 and 4 and are illustrated in Figures S3 and S4.

The non-eclipse case for satellite G01 and the yaw-steering case for satellites I03, I05 and M03 are shown in Figure S3. Figure S3(a) presents the observed-minus-calculated residuals for satellite G01 during the out-of-eclipse season, and Figure S3(b–d) shows the observed-minus-calculated residuals for satellites I03, I05 and M03 during the yaw-steering period. Among 200 days of SLR residual analysis, the ECOM + APR solution corresponds to the smaller observed-minus-calculated SLR residuals and the more stable SLR residuals compared with the ECOM-only solution. The extents to which the daily results for the SLR residuals are improved compared with the ECOM-only solution differ for the different cases. For the IGSO and MEO satellites, in the ECOM + APR solution, the SLR RMS residuals are improved by approximately 20–25 percent with respect to the ECOM-only solution during the yaw-steering period. For the G01 satellite, an 18 percent improvement during the non-eclipse season is achieved.

The eclipse case for satellite G01 and the yaw-fixed case for satellites I03, I05 and M03 are shown in Figure S4. Figure S4(a) presents the observed-minus-calculated residuals for satellite G01 during the eclipse season, and Figure S4(b–d) shows the observed-minus-calculated residuals for satellites I03, I05, and M03 in the yaw-fixed mode. During the yaw-fixed period, the ECOM + APR solution yields significant smaller observed-minus-calculated SLR residuals and more stable SLR residuals compared with the ECOM-only solution. Significant improvements compared with the ECOM-only solution are achieved in the daily results for the SLR residuals. For the IGSO and MEO satellites, in the ECOM + APR solution, the SLR RMS residuals improve by approximately 40 percent with respect to the ECOM-only solution during the yaw-fixed period. For the G01 satellite, a 32 percent improvement during the eclipse season is achieved.

Tables 2, 3, 4 also indicate a remarkable reduction in the systematic SLR bias of the ECOM + APR solution compared with the ECOM-only solution.

### Variability of the empirical ECOM SRP parameters

To further investigate the performance of the SRP model developed in this study in enhancing ECOM for precise orbit determination for BeiDou satellites, the estimated empirical ECOM SRP D0 parameters in the ECOM-only and ECOM + APR solutions are presented in Figures S5 and S6. When applying the 5-parameter ECOM without any a priori model, we can evaluate the D0 coefficient, which effectively measures the mean acceleration in the (anti-)Sun direction throughout the orbit of the BeiDou satellite of interest. Figure S5 shows that in the ECOM-only solution, the estimated D0 values for the four BeiDou satellites vary by approximately 5–7 nm/s^2^, and a significant β-angle dependence of the estimated D0 parameters is also evident for all BeiDou satellites. Figure S6 shows that in the ECOM + APR solution, with the introduction of the SRP model developed in this study as an a priori model to enhance ECOM, the β-angle dependence of the estimated D0 parameters is obviously removed. In addition, the estimated D0 parameters vary from 0 to 6 nm/s^2^ for the GEO satellite and from 0 to 3 nm/s^2^ for the IGSO and MEO satellites; the relatively small magnitudes of these variations indicate that the analytical SRP model developed in this study is close to ideal, making it obviously advantageous for improving the orbital accuracy that can be achieved for the BeiDou satellites currently in orbit.

## Discussion

In this study, an analytical solar radiation model for the current BeiDou satellites of the GEO, IGSO and MEO types, named IGGBSPM, was preliminarily established based on reasonably precise knowledge of the BeiDou satellites’ shapes, masses, and attitude control modes and the optical properties of their surfaces. A ray-tracing method proposed by Dr. Ziebart was used as the basis for developing the model.

We calculated the BeiDou GEO/IGSO/MEO orbits via numerical integration using the SRP model developed in this study, and the integrated orbits were then compared with the precise ephemerides. The results show that the orbits generated through integration are quite consistent with the precise BeiDou satellite ephemerides, differing by approximately 5 m for GEO satellites and 2 m for IGSO and MEO satellites. A constant Y-bias force was proposed for BeiDou satellites operating in the yaw-fixed mode. The addition of this Y-bias force enabled significant improvement in the orbital accuracy achieved via orbit integration for BeiDou satellites during the yaw-fixed period. For the along-track component, improvements in accuracy of 26, 26, 21 and 18 percent were achieved for satellites G01, I03, I05 and M03, respectively.

The SRP model developed in this study was used to enhance the 5-parameter ECOM for precise orbit determination for BeiDou satellites. We generated two dynamic force solutions: the ECOM-only solution and the ECOM + APR solution. Observation data from 9 iGMAS, 45 MGEX and 9 BETS stations from Days of Year 117–305 during the year of 2014 were processed using the two solutions. Then, SLR residual analyses were separately conducted for the yaw-steering and yaw-fixed cases. The results show that for IGSO and MEO satellites, the ECOM + APR solution yields an improvement of approximately 20–25 percent over the ECOM-only solution during the yaw-steering period and an improvement of approximately 40 percent during the yaw-fixed period. For satellite G01, improvements of 18 and 32 percent were achieved during the out-of-eclipse season and the eclipse season, respectively. A remarkable reduction in the systematic SLR bias was also observed in the ECOM + APR solution. An investigation of the empirical ECOM SRP D0 parameters estimated from the two solutions indicated that the β-angle dependence present in the ECOM-only solution vanishes in the ECOM + APR solution. Among the investigated satellites, the estimated ECOM D0 parameters vary from 0 to 6 nm/s^2^ for the GEO satellite and from 0 to 3 nm/s^2^ for the IGSO and MEO satellites; these relatively small magnitudes indicate that IGGBSPM is a close-to-ideal analytical SRP model, which is obviously advantageous for improving the orbital accuracy that can be achieved for BeiDou satellites currently in orbit.

Despite the success of IGGBSPM for currently in-orbit BeiDou satellites, continued efforts regarding the development of high-fidelity SRP models will be required in the future. In addition, considering albedo and thermal re-radiation in SRP modelling may improve the model accuracy^33^,^34^,^35^. Moreover, research on the variations of the solar irradiance factor E may also be advantageous for improving the SRP model developed in this study. Finally, in future work, with the aid of long-term SLR observations for BeiDou satellites, research on the empirical model scaling factors can be performed to further improve the accuracy and usability of IGGBSPM.

## Additional Information

**How to cite this article**: Tan, B. *et al*. A new analytical solar radiation pressure model for current BeiDou satellites: IGGBSPM. *Sci. Rep.* **6**, 32967; doi: 10.1038/srep32967 (2016).

## Supplementary Material

Supplementary Information

## Figures and Tables

**Table 1:** RMS residuals for satellites G01, I03, I05 and M03 generated from the daily RMS.

BeiDou satellite	Radial (in metres)	Along-track (in metres)	Cross-track (in metres)
G01 (C01)	0.867961907	5.208132586	1.207320728
I03 (C08)	0.616898434	2.078507464	1.308024616
I05 (C10)	0.622858389	2.130657929	1.28456792
M03 (C11)	0.759513796	2.162881294	1.046708035

**Table 2:** SLR residuals of the precise orbit determination solutions for satellite G01 computed from the ECOM-only and ECOM + APR solutions, in units of centimetres.

SRP model	G01			
	Eclipse season		Out-of-eclipse season	
	Mean	RMS	Mean	RMS
ECOM-only	−23.01	24.26	−16.68	18.64
ECOM + APR	−14.96	16.53	−13.61	15.27
Improvement, percent	35	**32**	18	**18**

**Table 3:** SLR residuals of the precise orbit determination solutions for satellites I03 and I05 computed from the ECOM-only and ECOM + APR solutions, in unit of centimetres.

SRP model	I03				I05			
	Yaw-fixed		Yaw-steering		Yaw-fixed		Yaw-steering	
	Mean	RMS	Mean	RMS	Mean	RMS	Mean	RMS
ECOM-only	3.81	10.58	−3.23	5.96	−5.57	11.91	−2.54	6.55
ECOM + APR	2.38	6.52	−2.29	4.39	−2.92	6.49	−1.85	4.88
Improvement, percent	38	**38**	29	**26**	48	**46**	27	**25**

**Table 4:** SLR residuals of the precise orbit determination solutions for satellite M03 computed from the ECOM-only and ECOM + APR solutions, in units of centimetres.

SRP model	M03			
	Yaw-fixed		Yaw-steering	
	Mean	RMS	Mean	RMS
ECOM-only	−3.51	10.37	−1.64	6.18
ECOM + APR	−2.13	6.32	−1.25	4.88
Improvement, percent	39	**39**	24	**21**
