# Supplementary material for: A new analytical solar radiation pressure model for current BeiDou satellites: IGGBSPM
Source: Sci Rep. 2016 Sep 6;6:32967. doi: 10.1038/srep32967 (PMC5011712; doi:10.1038/srep32967)
Supplement: Supplementary Information [file srep32967-s1.pdf]

Supplementary information for:

Title: A new analytical solar radiation pressure model for current BeiDou satellites: IGGBSPM

Authors: Bingfeng Tan[1]*, Yunbin Yuan[1], Baocheng Zhang[1], Hou Ze Hsu[1] & Jikun Ou[1].

[1]State Key Laboratory of Geodesy and Earth's Dynamics, Institute of Geodesy and Geophysics, Wuhan 430077, China.

*Correspondence: Bingfeng Tan. Tel: 86-18602719861; E-mail: bingfengtan@whigg.ac.cn.

Supplementary Information:

Supplementary Note S1

Supplementary Figures S1-S6

Supplementary Tables S1-S5

Supplementary Note S1

Glossary of terms and abbreviations

APR	A Priori
AU	The astronomical unit (1.4959787066 * 1e8 km)
BDS	Chinese BeiDou Navigation Satellite System
BETS	Beidou Experimental Tracking Stations
CODE	Center for Orbit Determination in Europe
ECEF	Earth-centred, Earth-fixed
ECI	Earth-centred inertial
EPS angle	Angle between the Earth and Sun direction as seen from the satellites
GEO	Geostationary Orbit
GNSS	Global Navigation Satellite System
GSPM	GPS Solar Pressure Models
IGGBSPM	Institute of Geodesy and Geophysics Beidou Solar Pressure Model
iGMAS	International GNSS Monitoring & Assessment System
IGS	International Global Navigation Satellite System Service
IGSO	Inclined Geosynchronous Orbit
ILRS	International Laser Ranging Service
MGEX	The Multi-GNSS Experiment
MEO	Medium altitude Earth Orbit
QZSS	Japanese Quasi Zenith Satellite System
RMS	Root Mean Square
SLR	Satellite Laser ranging
SRP	Solar Radiation Pressure

Supplementary Figures S1-S6

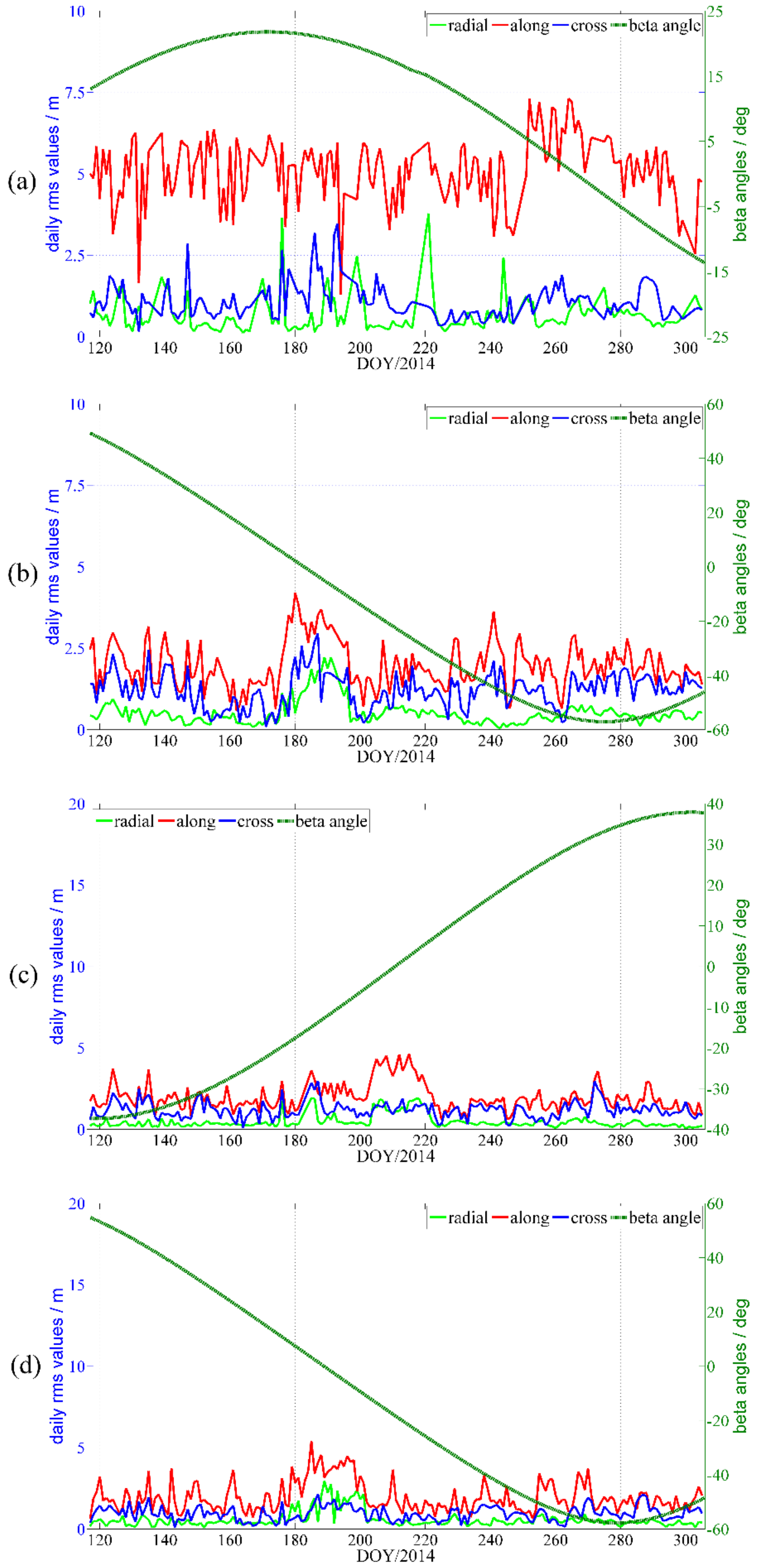

Figure S1: BeiDou satellite integration errors in the radial, along-track, and cross-track components. (a) Integration errors for BeiDou G01. (b) Integration errors for BeiDou I03. (c) Integration errors for BeiDou I05. (d) Integration errors for BeiDou M03.

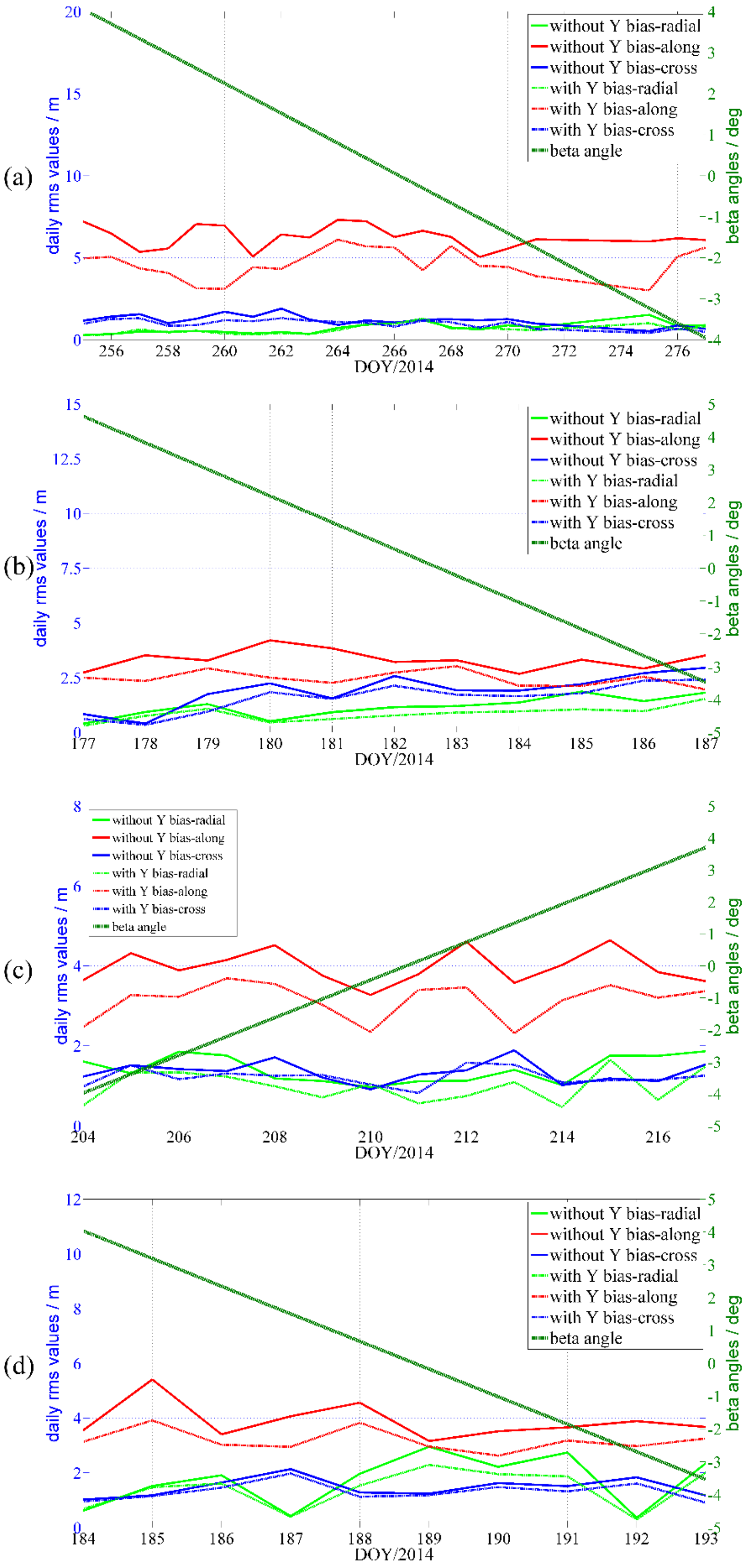

Figure S2: Integration errors in the radial, along-track, and cross-track components when the beta angle varies between -4 °and +4 °. (a) Integration errors for BeiDou G01. (b) Integration errors for BeiDou I03. (c) Integration errors for BeiDou I05. (d) Integration errors for BeiDou M03.

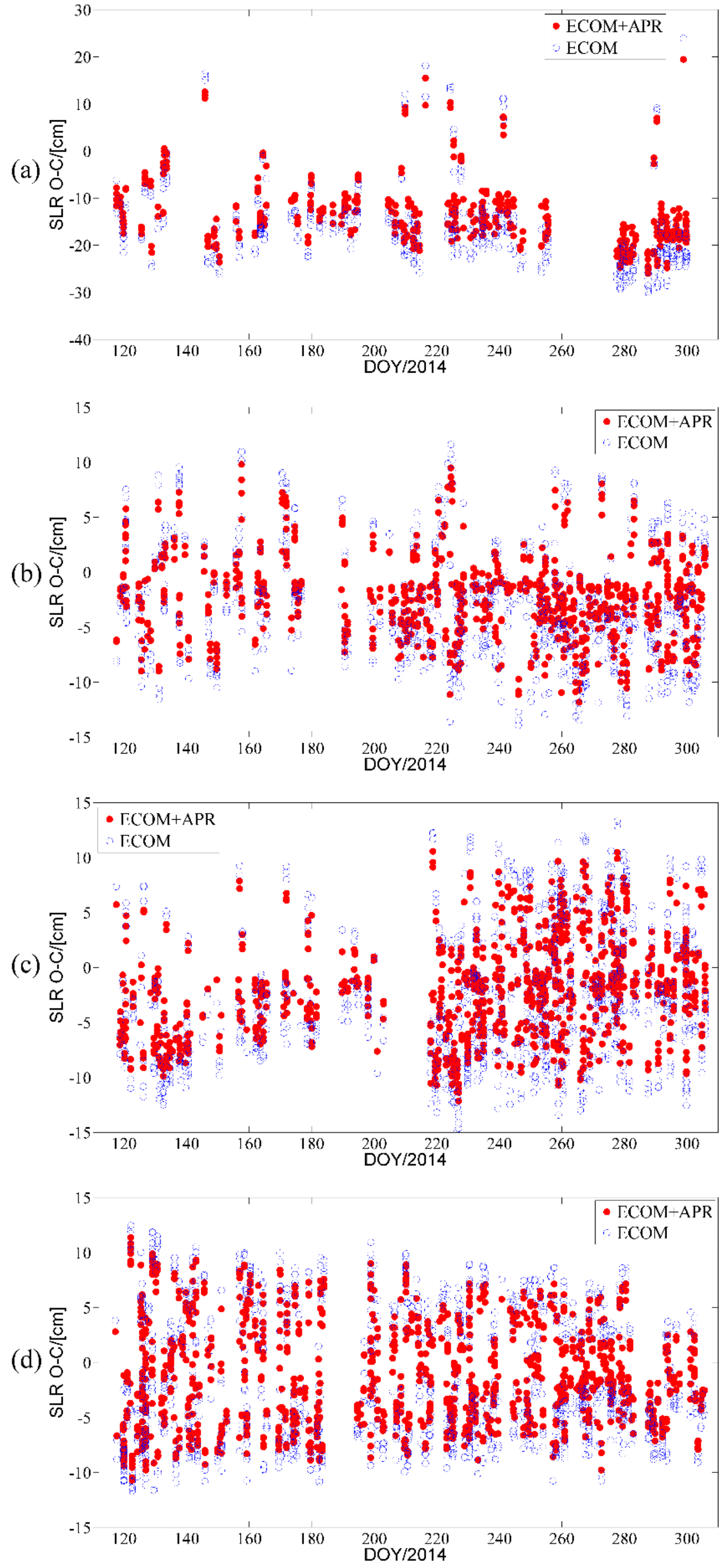

Figure S3: SLR validation for G01, I03, I05 and M03 when |β| is greater than 4°. (a) SLR validation for satellite G01 during the out-of-eclipse season. (b) SLR validation for satellite I03 during the yaw-steering period. (c) SLR validation for satellite I05 during the yaw-steering period. (d) SLR validation for satellite M03 during the yaw-steering period.

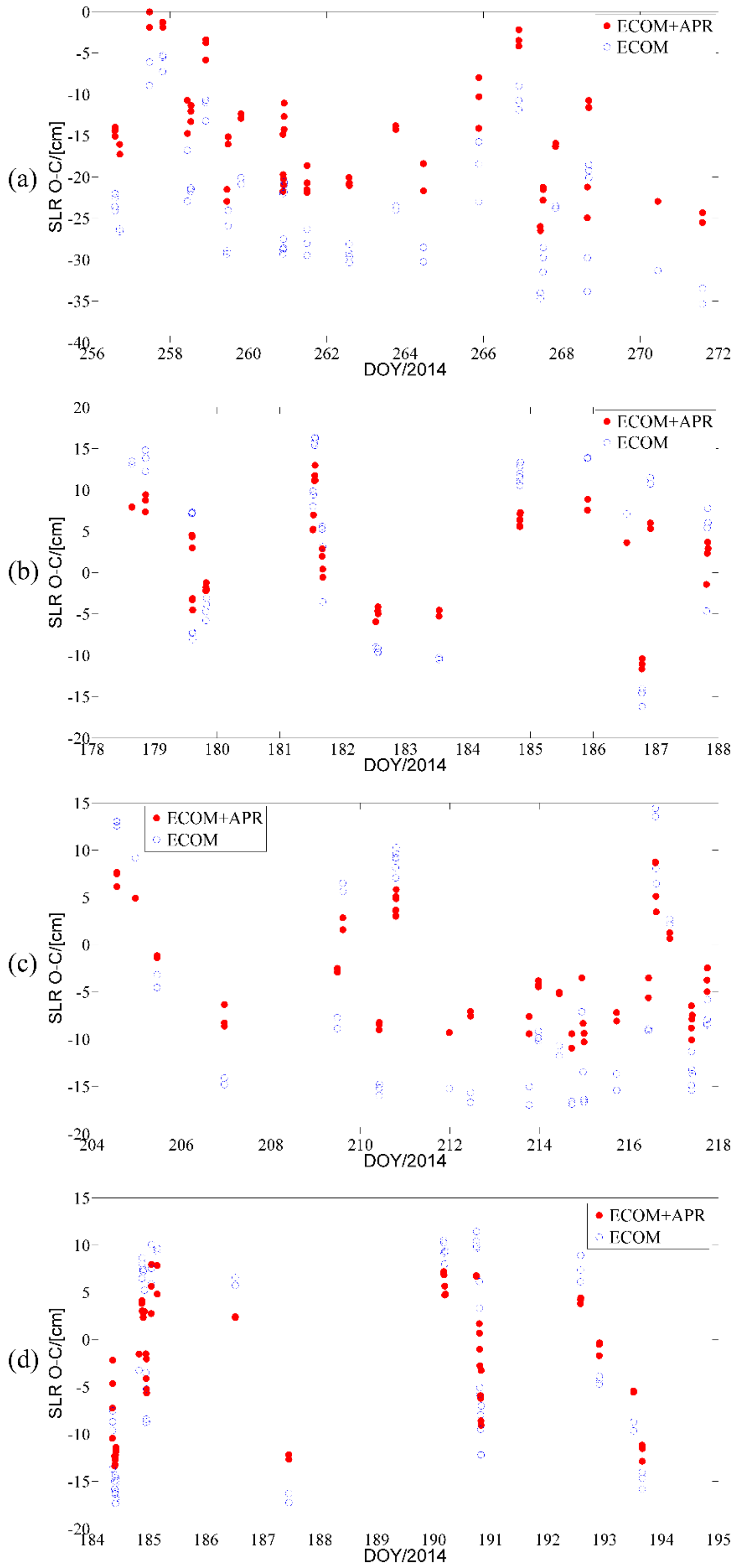

Figure S4: SLR validation for G01, I03, I05 and M03 when β varies between -4° and +4°. (a) SLR validation for satellite G01 during the eclipse season. (b) SLR validation for satellite I03 during the yaw-fixed period. (c) SLR validation for satellite I05 during the yaw-fixed period. (d) SLR validation for satellite M03 during the yaw-fixed period.

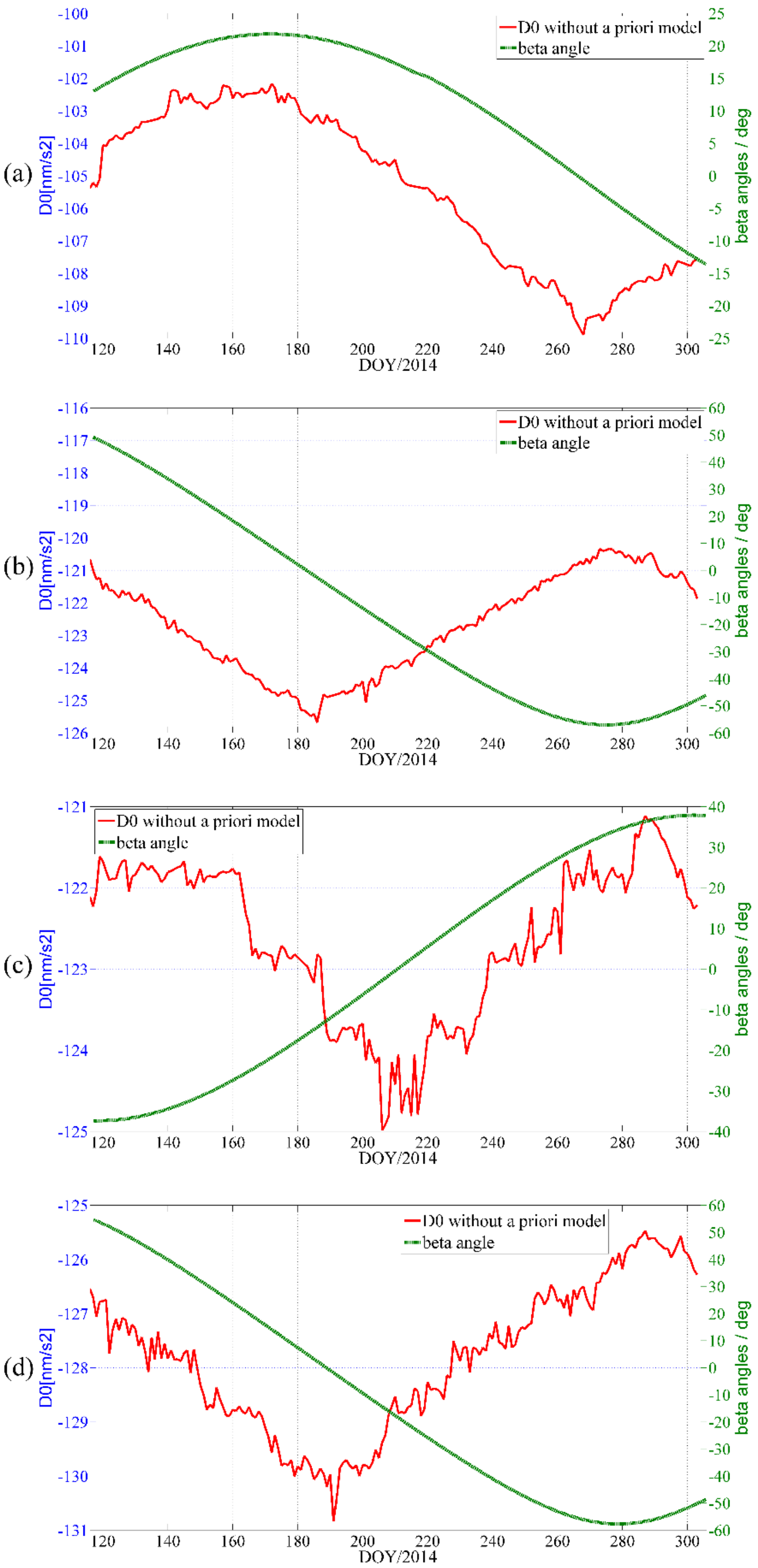

Figure S5: Variations in the SRP coefficients D_0 that are estimated when using the 5-parameter ECOM SRP model without IGGBSPM as an a priori model. (a) Variations in the D_0 coefficients estimated for G01. (b) Variations in the D_0 coefficients estimated for I03. (c) Variations in the D_0 coefficients estimated for I05. (d) Variations in the D_0 coefficients estimated for M03.

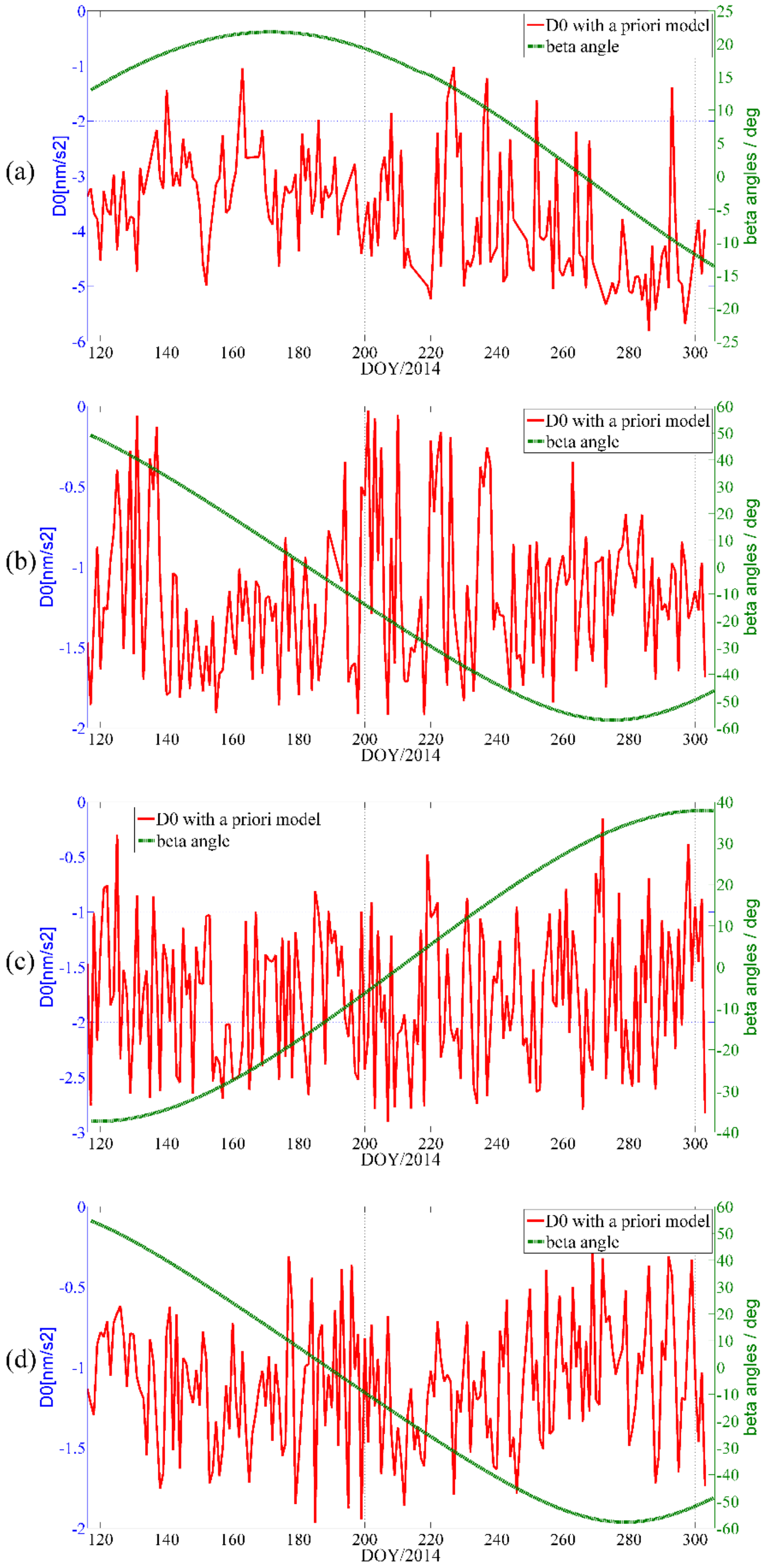

Figure S6: Variations in the SRP coefficients D_0 that are estimated when using the 5-parameter ECOM SRP model with IGGBSPM as an a priori model. (a) Variations in the D_0 coefficients estimated for G01. (b) Variations in the D_0 coefficients estimated for I03. (c) Variations in the D_0 coefficients estimated for I05. (d) Variations in the D_0 coefficients estimated for M03.

Supplementary Tables S1-S5

Table S1 BeiDou GEO IGGBSPM model coefficients for the accelerations along the X and Z axes

n	b(n), X axis	a(n), X axis	b(n), Z axis	a(n), Z axis
0	None	-3.26433E-10	None	-8.41536E-10
1	-1.16098E-07	-2.66402E-10	-3.28402E-10	-1.11579E-07
2	3.22251E-10	1.58843E-10	1.35017E-10	-3.8149E-10
3	1.21075E-09	8.92469E-11	-2.70806E-10	1.72286E-09
4	6.52259E-11	-1.20557E-11	1.34377E-12	1.31866E-10
5	4.98132E-10	2.48768E-11	-1.55643E-10	5.71202E-10
6	2.95846E-11	1.76225E-11	1.04017E-13	1.18175E-12
7	-3.50035E-11	1.21484E-11	-9.15862E-11	1.05111E-10
8	1.79728E-11	1.54374E-11	-7.15694E-12	2.93057E-11
9	1.00175E-10	7.96253E-12	-6.10223E-11	8.19033E-11
10	1.26857E-11	1.88113E-11	-1.03696E-11	-1.05333E-11
11	-3.20325E-11	1.14869E-11	-3.86818E-11	2.89449E-11
12	4.61716E-12	1.64371E-11	-6.68835E-12	8.23978E-12
13	5.08804E-11	8.61291E-12	-2.16028E-11	1.71284E-11
14	1.50445E-12	1.21342E-11	-3.10674E-13	-3.59121E-12
15	-2.77943E-11	4.03286E-12	-1.1662E-11	-9.92574E-12
16	1.85436E-12	8.69931E-12	-1.14942E-12	1.76433E-11
17	3.50137E-11	3.42063E-12	-6.57743E-12	-1.90331E-11

Table S2 BeiDou IGSO IGGBSPM model coefficients for the accelerations along the X and Z axes

n	b(n), X axis	a(n), X axis	b(n), Z axis	a(n), Z axis
0	None	-3.69329E-10	None	-9.76979E-10
1	-1.31651E-07	-2.8038E-10	-3.23022E-10	-1.26458E-07
2	3.76133E-10	1.68481E-10	1.59014E-10	-3.95607E-10
3	1.35784E-09	7.45993E-11	-3.27716E-10	2.00358E-09
4	8.17438E-11	5.03424E-12	1.61505E-11	1.34906E-10
5	5.75993E-10	2.75627E-11	-1.80853E-10	6.50451E-10
6	3.6495E-11	1.49441E-11	-4.32514E-12	-6.70874E-12
7	-2.96539E-11	1.21713E-11	-1.00123E-10	1.03749E-10
8	1.98666E-11	2.1758E-11	-1.06266E-11	3.60619E-11
9	1.12704E-10	9.83789E-12	-7.25782E-11	8.50548E-11
10	1.82062E-11	2.16496E-11	-1.11635E-11	-6.96836E-12
11	-4.02898E-11	1.57903E-11	-4.21013E-11	2.80921E-11
12	2.79189E-12	1.90711E-11	-5.29775E-12	9.64528E-12
13	5.84467E-11	8.54841E-12	-2.69207E-11	1.83541E-11
14	5.14884E-12	1.55239E-11	2.44777E-12	-6.35833E-12

15	-3.5496E-11	8.31354E-12	-1.18291E-11	-5.26509E-12
16	1.97078E-12	7.48586E-12	1.95389E-12	1.93695E-11
17	4.51046E-11	5.39716E-13	-5.37347E-12	-1.88543E-11

Table S3 BeiDou MEO IGGBSPM model coefficients for the accelerations along the X and Z axes

n	b(n), X axis	a(n), X axis	b(n), Z axis	a(n), Z axis
0	None	-3.8001E-10	None	-1.2821E-09
1	-1.36694E-07	-2.91087E-10	-3.34235E-10	-1.31305E-07
2	9.59312E-11	1.74319E-10	1.64728E-10	-5.76985E-10
3	1.25878E-09	7.64389E-11	-3.40236E-10	1.99665E-09
4	8.47739E-11	4.03437E-12	1.7188E-11	1.26865E-10
5	6.31705E-10	3.07834E-11	-1.87888E-10	6.87622E-10
6	3.85478E-11	1.38942E-11	-2.56494E-12	1.86459E-14
7	-5.02647E-11	1.25476E-11	-1.03301E-10	1.03168E-10
8	1.85098E-11	2.25703E-11	-9.17742E-12	3.41955E-11
9	1.29419E-10	1.0433E-11	-7.57139E-11	9.10657E-11
10	1.73498E-11	2.33906E-11	-9.52643E-12	-3.7953E-12
11	-4.94755E-11	1.59032E-11	-4.28249E-11	2.80236E-11
12	2.7422E-12	1.88364E-11	-4.78997E-12	7.1249E-12
13	6.80455E-11	8.08061E-12	-2.7695E-11	2.16166E-11
14	3.21257E-12	1.71643E-11	2.38677E-12	-4.07948E-12
15	-4.25251E-11	8.15562E-12	-1.25758E-11	-6.08878E-12
16	-1.02913E-13	6.68392E-12	1.24727E-12	1.76312E-11
17	5.206E-11	9.73186E-13	-5.94535E-12	-1.7888E-11

Table S4 Dynamic models used for integration

Geopotential	EGM2008 up to 12 × 12
Solid Earth tides	IERS Conventions 2010
Ocean tides	IERS Conventions 2010
Solid Earth pole tides	IERS Conventions 2010
N body	JPL DE405 ephemeris
SRP model	IGGBSPM developed in this study
Albedo, thermal re-radiation	Not included in the dynamic model
Y-bias	Not included in yaw-steering mode, included in yaw-fixed mode

Table S5 Summary of the precise orbit determination strategy for BeiDou satellites

Observations	Double-differenced ionosphere-free code and phase combination of B1 and B2
Elevation angle cut-off	7 °
Sampling rate	30 s
Data span	3 days

Station coordinates and ERPs	Fixed using estimates from the GPS daily POD process
Weighting	4 mm for phase observations, 2 m for code observations and elevation-dependent weighting
Satellite antenna PCO and PCV	Default PCO values for all BeiDou satellites and no PCV applied
Attitude model	Yaw-steering and yaw-fixed attitude modes
Ground antenna PCO and PCV	Not applied
Troposphere	Dry and wet GMF mapping functions; ZTDs estimated for each station at intervals of 2 hours; GPS+GLONASS daily ZTDs used as a priori values
Precession and nutation	IAU 2010 precession and IAU 2010 nutation model
Geopotential	EGM2008 12*12
Solid Earth tides, ocean tides and solid Earth pole tides	IERS Conventions 2010
N body	JPL DE405 ephemeris
SRP model	Two solutions: ECOM only and ECOM with the SRP model developed in this study as an a priori model
Orbital parameters	6 orbit elements and 5 ECOM SRP parameters were estimated:<br>constants in the D, Y and B directions; periodic terms in the B direction
Pseudo-stochastic orbit parameters	Every 12 hours; constrained to 1E-6 m/sec in the radial direction, 1E-5 m/sec in the along-track direction, and 1E-8 m/sec in the cross-track direction
Ambiguity	Real constant value for each ambiguity arc
